# Safety and Efficacy of Nemonoxacin vs Levofloxacin in Patients With Community-Acquired Pneumonia: A Systematic Review and Meta-Analysis of Randomized Control Trials

**DOI:** 10.7759/cureus.37650

**Published:** 2023-04-16

**Authors:** Alina S Khan, Arham Iqbal, Alina A Muhammad, Fariha Mazhar, Muniba F Lodhi, Komal F Ahmed, Satesh Kumar, Giustino Varrassi, Mahima Khatri

**Affiliations:** 1 Medicine and Surgery, Liaquat National Hospital and Medical College, Karachi, PAK; 2 Medicine and Surgery, Dow University of Health Sciences, Dow International Medical College, Karachi, PAK; 3 Medicine and Surgery, Ziauddin University, Karachi, PAK; 4 Medicine and Surgery, Shaheed Mohtarma Benazir Bhutto Medical College, Karachi, PAK; 5 Pain Medicine, Paolo Procacci Foundation, Rome, ITA; 6 Medicine and Surgery, Dow University of Health Sciences, Karachi, Karachi, PAK

**Keywords:** community-acquired pneumonia (cap), levofloxacin, nemonoxacin, pneumonia, quinolones, randomized control trials, safety and efficacy, systematic review and meta-analysis, teaes

## Abstract

Community-acquired pneumonia is a leading cause of morbidity and mortality throughout the world, which incurs significant healthcare costs. The aim of his meta-analysis is to assess the clinical efficacy and safety of a novel non-fluorinated quinolone, nemonoxacin, compared with levofloxacin in treating community-acquired pneumonia (CAP). A recursive literature search was conducted using PubMed, Google Scholar, and Scopus up to August 2022. All randomized clinical trials comparing nemonoxacin to levofloxacin for community-acquired pneumonia were included. The patients selected for this study had mild to moderate CAP. Each individual received treatment with either nemonoxacin (500 mg or 750 mg) or levofloxacin (500 mg) for a duration of 3-10 days. Four randomized control trials with a total of 1955 patients were included. Nemonoxacin and levofloxacin were found to have similar clinical cure rates in the treatment of CAP. There were no significant differences reported in the treatment-emergent adverse events between the two drugs (RR=0.95, 95% CI: 0.86, 1.08, I^2^=0%). However, the most frequent symptoms exhibited were gastrointestinal system-related. Both the dosages (500 mg and 750 mg) of nemonoxacin were found to have similar efficacy as that of levofloxacin. Our meta-analysis indicates that nemonoxacin is a well-tolerated and effective antibiotic therapy for the treatment of community-acquired pneumonia (CAP), with clinical success rates comparable to those of levofloxacin. Furthermore, the adverse effects associated with nemonoxacin are generally mild. Therefore, both the 500 mg and 750 mg dosages of nemonoxacin can be recommended as appropriate antibiotic therapy regimens for the treatment of CAP.

## Introduction and background

Community-acquired pneumonia (CAP) is defined as pneumonia that is acquired outside the hospital or extended-care facilities. It is the leading cause of pneumonia-related morbidity and mortality among all age groups worldwide [1,2]. A variety of pathogens, such as Streptococcus pneumoniae, Hemophilus influenza, atypical bacteria (i.e., Chlamydia pneumoniae, Mycoplasma pneumoniae, Legionella species), and viruses, may be responsible for community-acquired pneumonia [3]. Globally, 3-4 million people are affected by community-acquired pneumonia with high morbidity and mortality [4]. According to the recent report by the WHO Global Burden of Disease, lower respiratory tract infections (LRTIs), including CAP, cause approximately 429.2 million episodes of illness worldwide. Over the last few years, the emergence and transmission of antibiotic-resistant pathogens have become a considerable predicament in the clinical management of CAP. The Global Point Prevalence Survey (Global-PPS) reported that globally the most recurrent infection for which antibiotics were prescribed was pneumonia, accounting for about 19% of all the patients being treated [5]. Physicians are therefore advised to consider the factors that can aggravate the symptoms of pneumonia, such as the presence of comorbidities, severity of the disease, possibly perilous adverse effects of the drug, and antibiotic resistance, before prescribing an antimicrobial therapy for the treatment of CAP. The current Infectious Diseases Society of America/American Thoracic Society guidelines emphasizes monotherapy with a respiratory fluoroquinolone as an appropriate empirical treatment for adult CAP outpatients with cardiopulmonary disease or comorbidities [6]

Notwithstanding their effectiveness, quinolones are often associated with various gastrointestinal and neurological adverse effects, which significantly amplify the safety concerns surrounding their use [7].

Nemonoxacin, a novel non-fluorinated C-8 methoxy quinolone which targets DNA gyrase and topoisomerase IV, exhibits potent in vitro and in vivo activities against community-acquired pneumonia pathogens, including multidrug-resistant Streptococcus pneumoniae, penicillin-resistant Streptococcus pneumoniae, methicillin-resistant Staphylococcus aureus (MRSA), and ertapenem-non-susceptible Enterobacteriaceae [8-10]. The reduced incidence of toxic adverse effects is linked to the absence of fluorine moiety from nemonoxacin's quinolone structure [11]. Levofloxacin is a fluoroquinolone with broad-spectrum activity against several bacterial pathogens that cause CAP, including gram-positive and gram-negative bacteria, as well as antibiotic-resistant pathogens. It has proven to be an effective agent against penicillin-sensitive and penicillin-resistant Streptococcus pneumonia, which is by far the main causative agent of CAP [12]. Unlike levofloxacin, an additional characteristic of nemonoxacin is that it has shown poor activity against Mycobacterium tuberculosis (TB), multidrug-resistant TB, and non-multidrug-resistant TB [13]. Thus, its use would not mask or delay the diagnosis of TB [14]. In 2010, a study was done in Taiwan, China, and South Africa that investigated the efficacy and safety of nemonoxacin compared to that of levofloxacin in CAP outpatients. It was reported that nemonoxacin was not inferior to levofloxacin in either the evaluable intent-to-treat population or the clinical per protocol (PPc) population [15-17]. Although both drugs have been established as effective treatments for CAP, the safety and efficacy of nemonoxacin compared with levofloxacin remain controversial. Both drugs have favorable outcomes in treating the notorious illness, CAP. Unfortunately, despite the beneficial effects, these drugs come with their personalized set of adverse outcomes.

Few studies have compared the safety and efficacy of nemonoxacin and levofloxacin [14-17]. As a consequence, there is a limited comparative profile of the potency and effectiveness of these drugs. To analyze the safety and efficacy, a larger sample size along with variable outcomes is required. Because the studies are conducted in heterogeneous populations, therefore sample size is not adequate to explicate the desired concerns. Therefore, we analyzed the recent results to find a comprehensive overview of the safety and efficacy of nemonoxacin versus levofloxacin in the treatment of CAP. This is the first recently updated meta-analysis to the best of our knowledge.

## Review

Materials and method

This systematic review and meta-analysis were conducted by Preferred Reporting Items for Systematic Reviews and Meta-Analyses (PRISMA) guidelines [18]. An institutional review board (IRB) approval was not sought for this study as the data was publicly available.

Search Strategy

To retrieve all relevant articles, a literature review was conducted from inception to 13th February 2023 on PubMed, Google Scholar, and Scopus using a formulated search string. The search string was constructed based on different criteria of this study, using and combining key terms such as community-acquired pneumonia, pneumonia, CAP, nemonoxacin, quinolone, non-fluorinated quinolone, levofloxacin, ofloxacin, which resulted in the formation of the following Medical Subject Heading (MeSH) terms [[[nemonoxacin] OR [quinolone]] OR [non-fluorinated quinolone]] AND [[levofloxacin] OR [ofloxacin]]] AND [[[community-acquired pneumonia] OR [pneumonia]] OR [CAP]]. The detailed search strategy is given in the table in the appendix. The search string compared the safety and efficacy of the two quinolone drugs, namely nemonoxacin (given in two different dosages, 500 mg and 750 mg) versus levofloxacin (500 mg) administered to patients [who also had several different underlying disorders] suffering from community-acquired pneumonia. All articles were then transferred to EndNote™ X7 for the removal of duplicate studies. Two independent reviewers (AS and AI) screened the remaining articles based on the title and abstract before conducting a full-text screening. A third reviewer (AA) was consulted in the case of disparities. Studies were initially shortlisted based on title and abstract, after which the full text was assessed for eligibility. The references of the selected studies were also reviewed thoroughly.

Eligibility Criteria

Inclusion criteria: The meta-analysis included studies that met specific criteria. Firstly, the studies must have been published in English and have full text readily available for review. Secondly, all studies had to be randomized control trials (RCT), with patients of any age, ethnicity, and body mass index suffering from community-acquired pneumonia and any of the following underlying disorders: hypertension, diabetes mellitus, chronic obstructive pulmonary disease (COPD), chronic bronchitis (CB), hepatitis B, hyperlipidemia, allergic rhinitis, and tuberculosis. Thirdly, the studies had to involve treatment with quinolones, namely nemonoxacin and levofloxacin, with different dosages of 500 mg and 750 mg for nemonoxacin and 500 mg for levofloxacin. The studies assessed common treatment-emergent adverse events (TEAEs) such as ventral nervous system (CNS), red blood cells (RBCs), white blood cells (WBCs), platelets, gastrointestinal tract (GIT), cardiovascular and liver enzymes, and drug-related TEAEs such as RBCs, WBCs, platelets, and gastrointestinal tract. Additionally, the studies underwent a comprehensive evaluation to ascertain whether they provided sufficient information on each drug group's safety, efficacy, and side effect profile independently.

Exclusion criteria: Certain types of studies were excluded to ensure the quality of the meta-analysis. These included reviews, editorials, protocols, case reports, and studies without comparison and outcomes. Only studies that had a placebo and control group were included. Duplicates of previous publications should have been considered. In addition, studies that did not provide sufficient data for estimating a mean difference (MD) with a 95% confidence interval (CI) were excluded from the meta-analysis. These exclusion criteria were implemented to ensure the validity and reliability of the findings in the meta-analysis.

*Data Extraction* 

The studies done by the data extraction team (AS and AI) that were to be included in the meta-analysis had the first author's name, the year in which the relevant randomized control trial was published, type and phase of the RCT trial, duration of RCT trial study, race and ethnicity, underlying comorbidities, dosages of drugs administered, the total number of patients included in the study, and several patients in individual groups (nemonoxacin 500 mg and 750 mg) and levofloxacin (750 mg). Outcomes of interest include common and drug-related TEAEs. Common TEAEs can be defined as neutropenia, thrombocytopenia, diarrhea, nausea, vomiting, stomach upset/abdominal discomfort, abnormal liver functions, elevated serum aspartate transaminase (AST), elevated alanine transaminase (ALT), QT prolongation, headache, skin rash, dizziness, and leukopenia. Drug-related TEAEs are defined as neutropenia, thrombocytopenia, anemia, diarrhea, nausea, and headache.

Quality Assessment and Risk of Bias

The Cochrane method was used for the evaluation of selected RCTs.

*Statistical Analysis* 

Only comparative studies were analyzed statistically using Review Manager 5.4.1 (The Nordic Cochrane Centre, Denmark) and comprehensive meta-analysis. This meta-analysis provides a pooled effect of relative risks (RRs) for dichotomous outcomes and weighted mean differences (WMDs) for continuous outcomes calculated utilizing the generic-inverse variance with a random-effects model. Forest plots were used to display the results of pooled analyses. To assess publication bias, funnel plots were constructed for the two main outcomes. Low (25%), moderate (25-75%), and high (>75%) levels of heterogeneity were determined using Higgin's I^2^ test. All analyses were considered significant if the p-value was less than 0.05.

Since the data was compiled and synthesized from earlier clinical trials for which the researchers had already received informed consent, no ethics committee approval was required for this study.

Results

Study Selection

**Figure 1:**
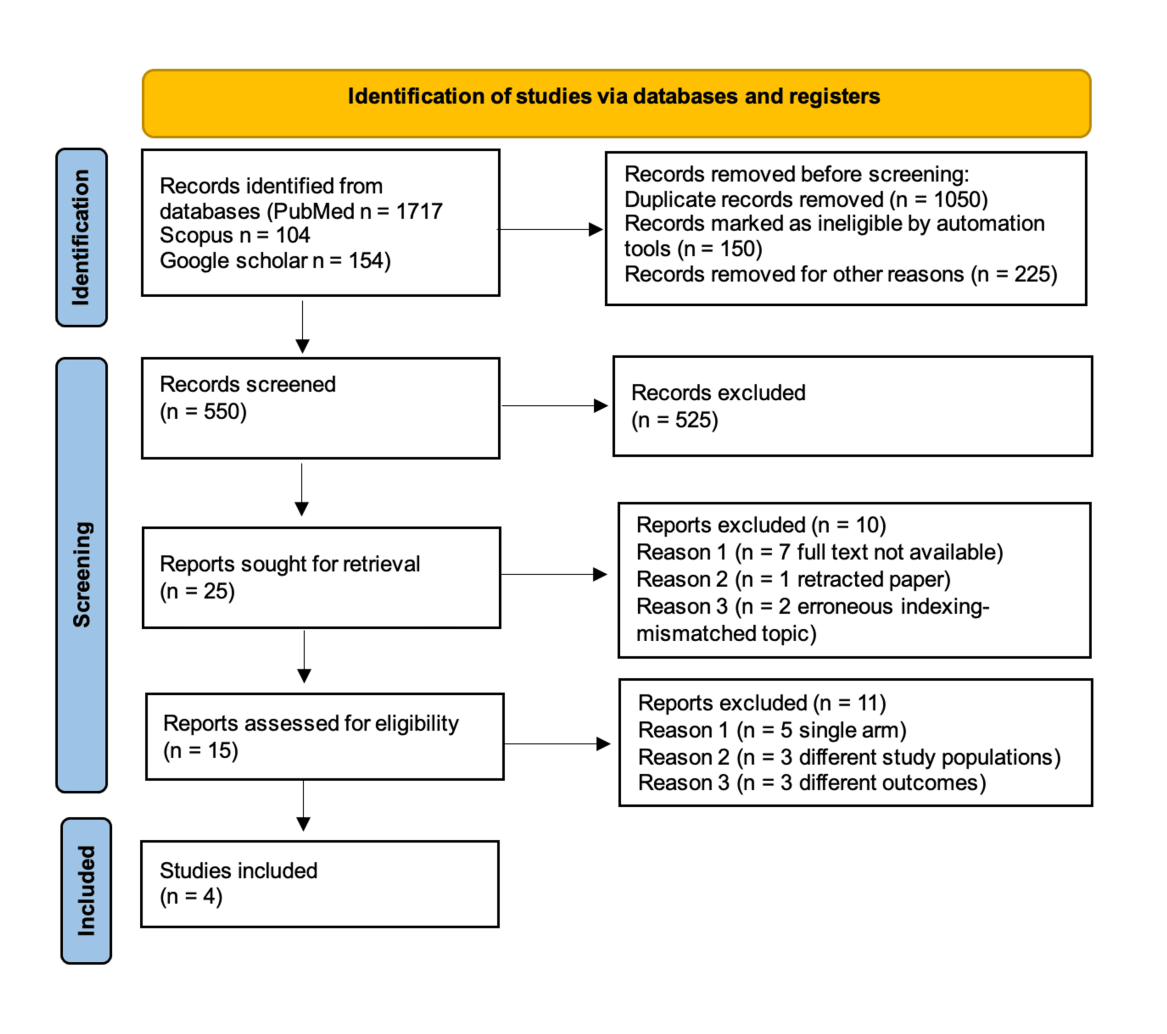
Preferred Reporting Items for Systematic Reviews and Meta-Analyses (PRISMA) flowchart

By applying the search strategy, an extensive search was conducted on three databases (PubMed, Google Scholar, and Scopus), which yielded a total of 1975 results. Implementing the inclusion and exclusion criteria, a total of four studies were excluded in the process of title and abstract screening. The remaining records then underwent full-text review; at last, only four randomized controlled trials that were deemed fit according to our inclusion criteria were inducted for this meta-analysis. The selection process is summarized in Figure 1.

Baseline Characteristics

The selected four RCTs included patients with community-acquired pneumonia, with several underlying diseases (such as hypertension [HTN], DM, COPD, CB, hepatitis B, hyperlipidemia, allergic rhinitis, and tuberculosis), with 1322 patients receiving nemonoxacin (500 mg and 750 mg) and 633 patients receiving levofloxacin (500 mg). The characteristics of the individual studies are summarized in the tables in the appendix. At least two outcomes, namely common TEAEs (sub-classified into the cardiovascular system (CVS), CNS, GIT, liver enzymes, and dermatology) and drug-related TEAEs (GIT and derma), were evaluated in the four studies involving CAP treated with the aforementioned quinolones. The patients participating in each trial were orally administered both quinolones. In three [14-17] of the four studies, three different dosages (nemonoxacin 500 mg, 750 mg, and levofloxacin 750 mg) were administered, whereas, in one [16], only two dosages (nemonoxacin 500 mg and levofloxacin 750 mg) were administered. None of the studies included a control group.

Regarding the phases, Van Rensburg et al. [14] study was in the second phase, whereas Liu et al. [15] and Yuan et al. [16] studies were in the third phase trials. Finally, Cheng et al. [17] study is a mixture of the second and third phase trial. Regarding the time duration for the RCT, Van Rensburg et al. [14] study was seven days, whereas Liu et al. [15] and Yuan et al. [16] studies were done in 7-10 days. Lastly, Cheng et al. [17] study was done in 3-10 days. As indicated by the research type, Table 1 provides information on the baseline characteristics of the included patients.

**Table 1:** Baseline characteristics of patients [14-17] RCT- randomized controlled trial; SD - standard deviation

Study	Study design	Total number of patients	Patients on nemonoxacin	Patients on levofloxacin	BMI (mean ± SD)		Age (mean ± SD)		Gender (mean ± SD)			
									Male		Female	
					Nemonoxacin	Levofloxacin	Nemonoxacin	Levofloxacin	Nemonoxacin	Levofloxacin	Nemonoxacin	Levofloxacin
Van Rensburg et al. (2010) [14]	RCT	265	175	90	23.95 ± 6.25	23.4 ± 4.9	44 ± 50.25	44.5 ± 16.4	44 ± 50.25	55 ± 61.1	43.5 ± 49.75	35 ± 38.9
Liu et al. (2015) [15]	RCT	192	116	52	22.55 ± 3.3	22.2 ± 3.4	38.4 ± 14.9	39.7 ± 15.1	N/A	N/A	N/A	N/A
Yuan et al. (2017) [16]	RCT	527	356	171	22.7 ± 3.2	23.0 ± 3.0	43.6 ± 14.9	43.6 ± 14.5	169 ± 51.5	N/A	N/A	159 ± 48.5
Cheng et al. (2018) [17]	RCT	995	675	320	23.2 ± 4.63	23.0 ± 3.71	41.5 ± 15.495	42.8 ± 15.35	181.5 ± 52.35	188 ± 58.8	157 ± 47.65	132 ± 41.3

Quality Assessment and Publication Bias

**Figure 2:**
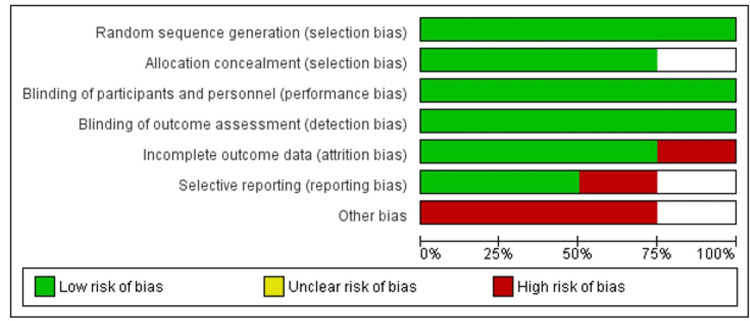
Quality assessment for RCTs using the Cochrane risk of bias tool RCTs- randomized controlled trials

**Figure 3:**
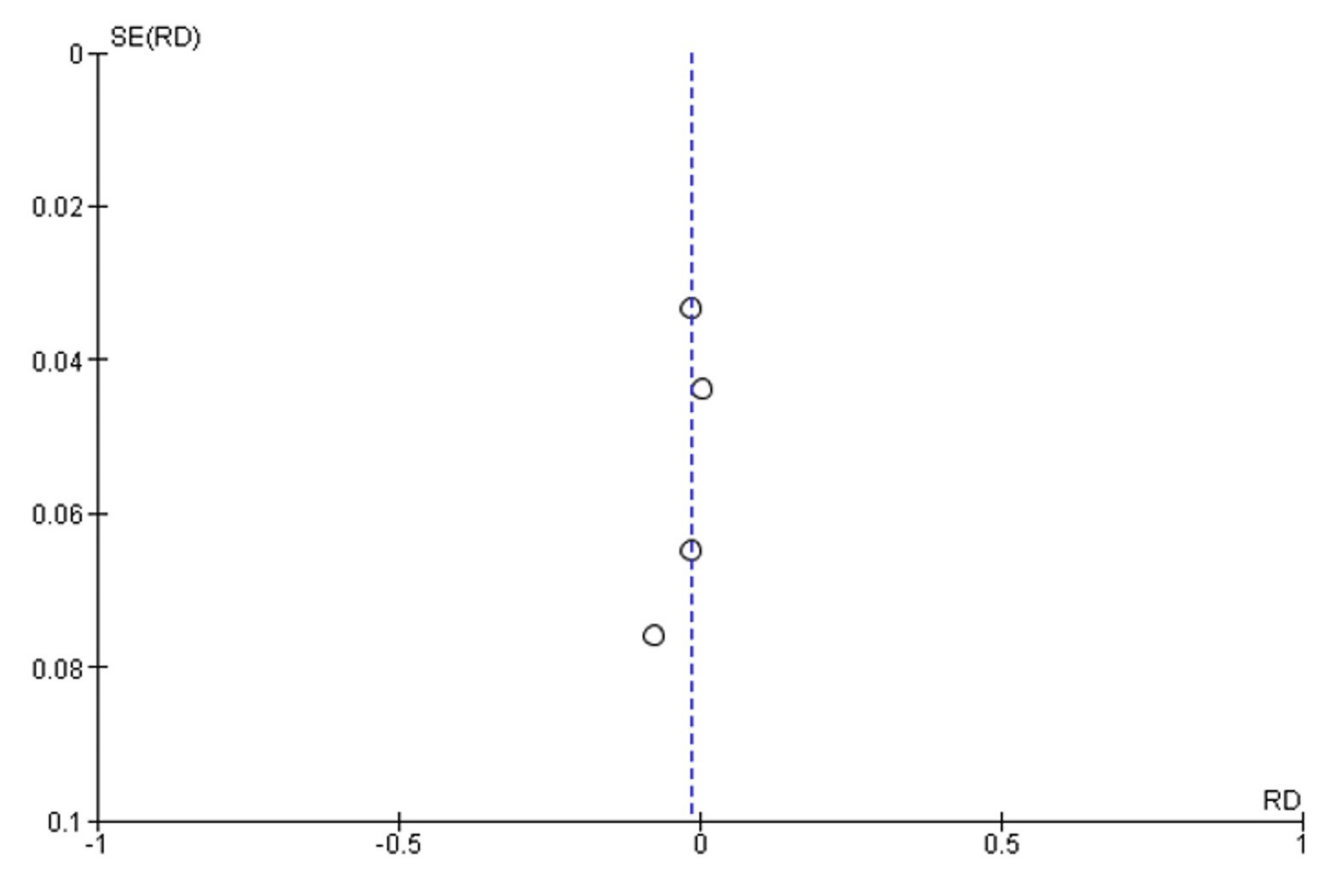
Funnel plot of common treatment-emergent adverse events (TEAEs) SE - standard error; RD - risk difference

**Figure 4:**
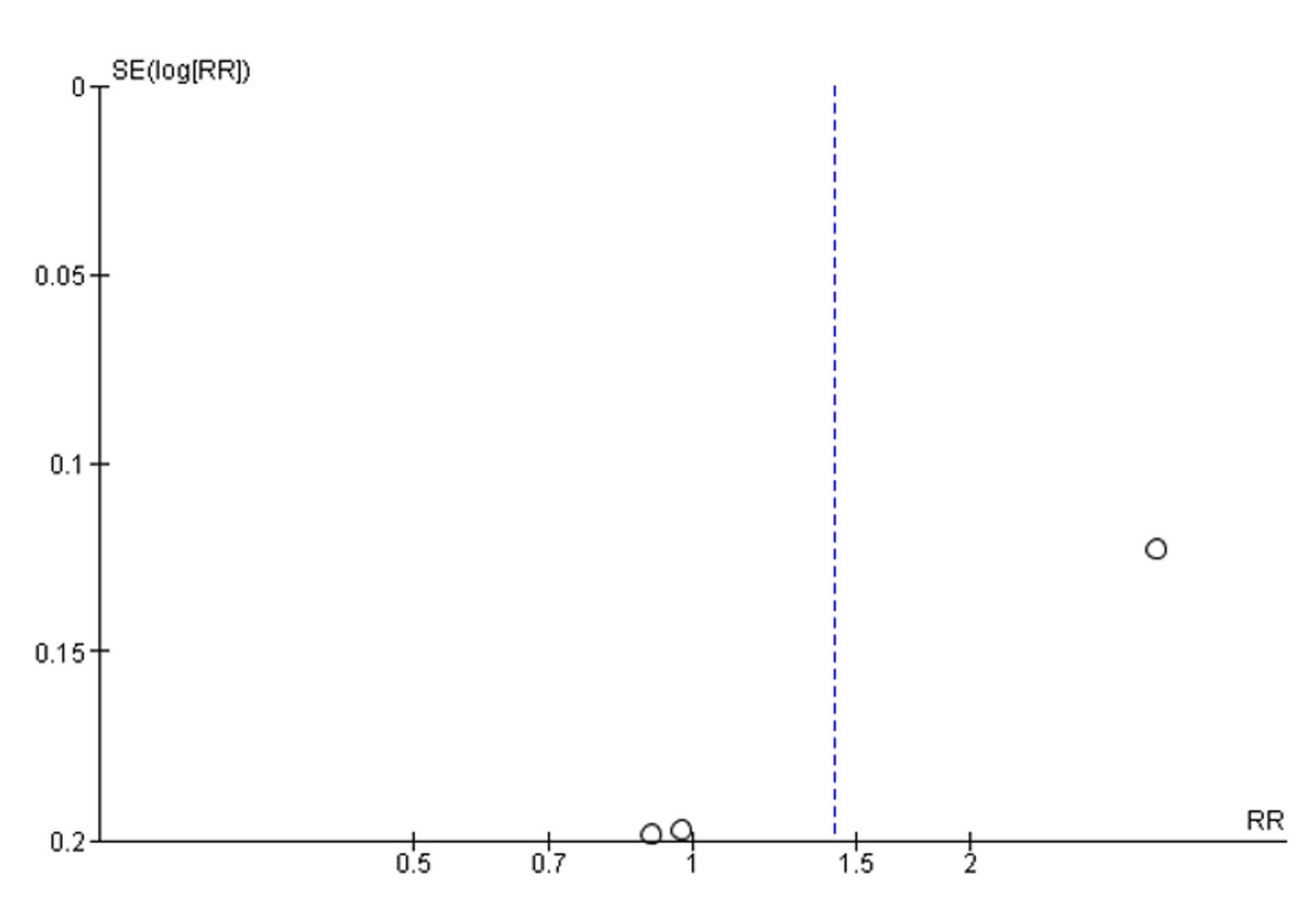
Funnel plot of drug-related treatment-emergent adverse events (TEAEs) SE - standard error; RR - relative risk

Quality assessment of all the RCTs was done by using the Cochrane Collaboration tool for assessing the risk of bias in randomized trials, which showed that Yuan et al. [16] study was found to be low-risk in selection bias, performance bias, detection bias, and attrition bias. Liu et al. [15] study was found to be low-risk in selection bias, performance bias, detection bias, attrition bias as well as reporting bias. Cheng et al. [17] study was found to be low risk in selection bias, performance bias, detection bias, and attrition bias and was found to be high risk in reporting bias. Van Rensburg et al. [14] study was found to be low risk in selection bias, performance bias, detection bias, and reporting bias, although it was found to be high risk in attrition bias, as shown in Figure 2. Funnel plots of the main outcomes showed that this study has no publication bias, as shown in Figures 3 and 4.

Outcomes

All of the studies recruited in this research [14-17] exhibit a range of side effects of the drugs used. Although these studies have some differences, they share similar outcomes as well. Tables 2 and 3 summarize outcomes on an individual study basis.

**Table 2:** Common treatment-emergent adverse events (TEAEs) [14-17] SD - standard deviation; AST - aspartate transaminase; ALT - alanine transaminase

TEAEs	Van Rensburg et al. (2010) [14]			Liu et al. (2015) [15]			Yuan et al. (2017) [16]			Cheng et al. (2018) [17]		
	Nemonoxacin 500 mg (SD)	Nemonoxacin 750 mg (SD)	Levofloxacin 500 mg (SD)	Nemonoxacin 500 mg (SD)	Nemonoxacin 750 mg (SD)	Levofloxacin 500 mg (SD)	Nemonoxacin 500 mg (SD)	Nemonoxacin 750 mg (SD)	Levofloxacin 500 mg (SD)	Nemonoxacin 500 mg (SD)	Nemonoxacin 750 mg (SD)	Levofloxacin 500 mg (SD)
Neutropenia	9 ± 10.1	9 ± 10.5	10 ± 11.1	2 ± 3.2	2 ± 3.4	2 ± 3.6	N/A	N/A	N/A	23 ± 4.4	0	8 ± 2.5
Thrombocytopenia	2 ± 2.2	4 ± 4.7	2 ± 2.2	N/A	N/A	N/A	N/A	N/A	N/A	2 ± 2.2	4 ± 4.7	2 ± 2.2
Diarrhea	7 ± 7.9	4 ± 4.7	2 ± 2.2	N/A	N/A	N/A	N/A	N/A	N/A	7 ± 1.3	2 ± 1.3	3 ± 0.9
Nausea	1 ± 1.1	7 ± 8.1	3 ± 3.3	1 ± 1.6	6 ± 10.2	1 ± 1.8	12 ± 3.4	N/A	5 ± 2.9	13 ± 2.5	11 ± 7.1	8 ± 2.5
Vomiting	N/A	N/A	N/A	0	4 ± 6.8	2 ± 3.6	6 ± 1.7	N/A	5 ± 2.9	6 ± 1.2	N/A	7 ± 2.2
Stomach upset/abdominal pain	N/A	N/A	N/A	0	2 ± 3.4	1 ± 1.8	8 ± 2.2	N/A	1 ± 0.6	5 ± 1.0	2 ± 1.3	3 ± 0.9
Abnormal liver function	N/A	N/A	N/A	0	3 ± 5.1	0	N/A	N/A	N/A	0	4 ± 2.6	1 ± 0.3
Elevated AST	N/A	N/A	N/A	N/A	N/A	N/A	9 ± 2.5	N/A	4 ± 2.3	10 ± 1.9	1 ± 0.6	3 ± 0.9
Elevated ALT	N/A	N/A	N/A	3 ± 4.8	0	1 ± 1.8	21 ± 5.9	N/A	8 ± 4.7	23 ± 4.4	0	8 ± 2.5
QT interval prolongation	N/A	N/A	N/A	2 ± 3.2	3 ± 5.1	1 ± 1.8	N/A	N/A	N/A	4 ± 0.8	4 ± 2.6	5 ± 1.5
Headache	2 ± 2.2	4 ± 4.7	1 ± 1.1	N/A	N/A	N/A	N/A	N/A	N/A	5 ± 1.0	2 ± 1.3	3 ± 0.9
Dizziness	5 ± 5.6	5 ± 5.8	2 ± 2.2	N/A	N/A	N/A	10 ± 2.8	N/A	3 ± 1.8	10 ± 1.9	3 ± 1.9	3 ± 0.9
Leukopenia	N/A	N/A	N/A	4 ± 6.5	6 ± 10.2	4 ± 7.1	7 ± 2.0	N/A	3 ± 1.8	12 ± 2.3	7 ± 4.5	10 ± 3.1
Skin rash	N/A	N/A	N/A	N/A	N/A	N/A	2 ± 0.6	N/A	4 ± 2.3	2 ± 0.4	0	4 ± 1.3

**Table 3:** Drug-related treatment-emergent adverse events (TEAEs) [14-17] SD - standard deviation

TEAEs	Van Rensburg et al. (2010) [14]			Liu et al. (2015) [15]			Yuan et al. (2017) [16]			Cheng et al. (2018) [17]		
	Nemonoxacin 500 mg (SD)	Nemonoxacin 750 mg (SD)	Levofloxacin 500 mg (SD)	Nemonoxacin 500 mg (SD)	Nemonoxacin 750 mg (SD)	Levofloxacin 500 mg (SD)	Nemonoxacin 500 mg (SD)	Nemonoxacin 750 mg (SD)	Levofloxacin 500 mg (SD)	Nemonoxacin 500 mg (SD)	Nemonoxacin 750 mg (SD)	Levofloxacin 500 mg (SD)
Neutropenia	8 ± 9.0	8 ± 9.3	10 ± 11.1	N/A	N/A	N/A	N/A	N/A	N/A	13 ± 2.5	13 ± 8.4	14 ± 4.4
Thrombocytopenia	2 ± 2.2	4 ± 4.7	1 ± 1.1	N/A	N/A	N/A	N/A	N/A	N/A	4 ± 0.8	4 ± 2.6	2 ± 0.6
Diarrhea	N/A	1 ± 1.2	5 ± 5.6	N/A	N/A	N/A	N/A	N/A	N/A	7 ± 1.3	2 ± 1.3	3 ± 0.9
Nausea	1 ± 1.1	5 ± 5.8	3 ± 3.3	N/A	N/A	N/A	N/A	N/A	N/A	13 ± 2.5	11 ± 7.1	8 ± 2.5

Common TEAEs

**Figure 5:**
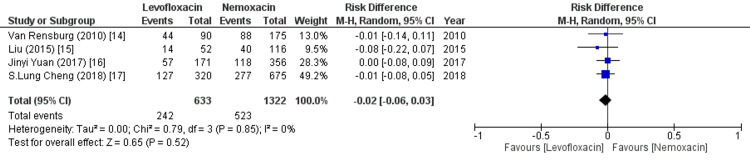
Forest plot of common treatment-emergent adverse events (TEAEs) RR - relative risk; CI - confidence interval

When we compare the safety and efficacy of the two drug regimens, levofloxacin has slightly less risk of inducing common TEAEs as compared to nemonoxacin (RR=0.96 [0.86,1.08], p=0.53, I^2^=0%) as shown in Figure 5. Overall, this adverse event is statistically insignificant as an outcome.

GIT-associated adverse events: All four recruited studies [14-17] reported nausea as a shared TEAE. The pooled analysis shows that treatment with levofloxacin is associated with a decreased risk of nausea compared to nemonoxacin (RR=0.71 [0.41, 1.22] p=0.22; I^2^=0%). Conversely, this value is statistically insignificant (p=0.22). Three out of four recruited studies [15-17] demonstrated the outcome of vomiting. Yet again, the treatment with levofloxacin proved to be associated with a decreased risk of vomiting when compared to nemonoxacin (RR=1.49 [0.76, 2.93], p=0.25, I^2^=0%); this outcome is statistically insignificant. Hence there appears to be no distinctive difference in outcomes overall between the two groups.

Diarrhea appeared to be a TEAE in two out of the four studies enlisted in our meta-analysis research [14,17]. We acquired similar results where levofloxacin had a lesser risk of inducing diarrhea than nemonoxacin (RR=0.52 [0.20, 1.39], p=0.19, I^2^=0%), but based on the p-value, this outcome is statistically insignificant. Lastly, there was a reduced risk of inducing an upset stomach with levofloxacin than with administering nemonoxacin, which was demonstrated in three out of four studies [15-17] (RR=0.69 [0.25, 1.93], p=0.48, I^2^=0%), but this TEAE warrants no statistically significant effect on the overall outcome.

Abnormal liver function test: Abnormal liver enzyme functions were reported by two out of four recruited studies [15,17]. The pooled analysis displayed that treatment with levofloxacin was associated with a decreased risk of TEAE, as compared to nemonoxacin. Overall there was no statistically significant difference between the two groups (RR=0.44 [0.08, 2.54], p=0.36, I^2^=0%). The adverse event of elevated AST was reported by two out of the four studies [16,17], and the outcome of elevated ALT emerged in three out of the four studies [15-17], also depicted insignificant values (RR=0.74 [0.32, 1.76], p=0.50, I^2^=0% and RR=0.76 [0.44, 1.31], p=0.33, I^2^=0%, respectively). The risk ratio values support the previously drawn conclusion that levofloxacin is less likely to cause elevated liver enzyme levels. However, based on the p-value, this TEAE is an insignificant outcome to be measured.

Hematological adverse events: The adverse events while using levofloxacin and nemonoxacin for the treatment of CAP were evaluated based on blood-related disorders, namely neutropenia, leukopenia, and thrombocytopenia. Neutropenia was reported by three out of four studies [14-17]. Our pooled analysis demonstrates (RR=1.11 [0.70, 1.76], p=0.65, I^2^=0%) that there was an increased risk of this outcome in patients using nemonoxacin compared to the other group but the overall result was statistically insignificant among the two drugs.

Leukopenia appeared to be a TEAE in three out of four studies [15-17], and our meta-analysis reveals (RR=1.01 [0.57, 1.78], p=0.97, I^2^=0%) that there is no significant statistical difference in both the groups. Two out of four publications reported thrombocytopenia-related TEAE [14,17]. The forest plot shows (RR=0.58 [0.19, 1.76], p=0.34) that the relative risk of this adverse event is less likely in patients using levofloxacin as compared to nemonoxacin. However, the overall analysis and the subsequent p-value determine that this outcome does not cause any significant difference between the two groups.

Cardiovascular adverse events: QT interval prolongation and bundle branch blockage were evaluated and reported in two out of four studies [15,17]. The QT interval prolongation TEAE plot (RR=1.04 [0.39, 2.79], p=0.93, I^2^=0%) can be interpreted as an insignificant difference between both groups. The bundle branch block outcome forest plot (RR=0.43 [0.05, 3.67], p=0.44, I^2^=0%) revealed the risk of this event in patients using nemonoxacin, although it was statistically insignificant.

CNS-related adverse events: To evaluate the outcomes of levofloxacin and nemonoxacin in patients with CAP, headache and dizziness were also taken under consideration. The pooled analysis revealed that only two out of four publications [14,15] were relevant to headache TEAE. The forest plot demonstrated significance (RR=0.67 [0.22, 2.08], p=0.49, I^2^=0%). Based on the relative risk comparing levofloxacin and nemonoxacin, levofloxacin has a reduced risk of a headache than nemonoxacin. The adverse event of dizziness was found in three out of four studies [14,16,17]. The TEAE plot showed that it was not significant (RR=0.50 [0.23, 1.08], p=0.08, I^2^=0%). The relative risk, while comparing levofloxacin with nemonoxacin, shows that levofloxacin had lesser incidences of dizziness compared to the other drug. The overall analysis based on the p-value of the two plots revealed that CNS-based effects in both groups are nearly the same and insignificant.

Skin-related TEAEs: The data on skin rash was reported in two out of four studies [16,17], and the pooled analysis revealed (RR=4.19 [1.27, 13.84], p=0.02, I^2^=0%) that there is a significantly increased risk of this particular adverse effect. Therefore, skin-related TEAEs were significantly reported in the group taking the levofloxacin regimen.

Drug-related TEAEs

**Figure 6:**
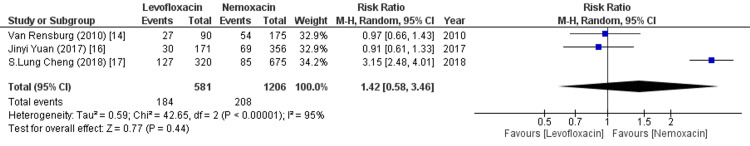
Forest plot of drug-related treatment-emergent adverse events (TEAEs) RR - relative risk, CI - confidence interval

Drug-related common TEAEs were reported in three out of the four papers [14,16,17]. Pooling of these studies demonstrated that the comparison of levofloxacin and nemonoxacin was not significantly causing any statistical difference between the two groups (RR=1.42 [0.58,3.46], p=0.44, I^2^=95%) as shown in Figure 6. Therefore, drug-related TEAEs in both groups are nearly the same. Moreover, based on the p-value, the overall effect of this outcome appears to be insignificant.

Drug-related hematological adverse events: To evaluate the influence of drug-related TEAEs blood disorders, the data was reported by two out of four studies [14,17]. The effect on blood-related disorders was examined by the following parameter group. However, this risk was insignificant based on the p-value deduced from the forest plot. For the thrombocytopenia plot (RR=0.44 [0.13, 1.54], p=0.20, I^2^ =0%), the pooled analysis revealed a low risk of this drug-related TEAE in the patients with the levofloxacin regimen as compared to nemonoxacin. There is no statistically significant difference between the two medications that are being compared as demonstrated by the p-value.** **Our meta-analysis demonstrated (RR=1.17 [0.72, 1.90], p=0.53, I^2^=0%) that there was an increased risk of neutropenia in the group being treated with nemonoxacin as compared to the other group. The pooled analysis revealed that there was a reduced risk of this drug-related TEAE in the patients on the levofloxacin regimen as compared to nemonoxacin. The overall analysis and the p-value show no statistically significant difference between the two drugs.

Drug-related GIT adverse events: The occurrence of drug-related TEAEs, namely nausea and diarrhea, was reported by two out of four studies [14,17]. The analysis demonstrated (RR=2.27 [0.17,29.96], p=0.53, I^2^=77%) an increased risk of diarrhea in patients taking nemonoxacin, but the p-value depicts this risk as statistically insignificant. Moreover, when we evaluated nausea as an outcome, it showed that the relative risk of this outcome was more in the group being treated with nemonoxacin (RR=0.76 [0.39, 1.51], p=0.44, I^2^=0%), but the overall analysis demonstrated that this risk is statistically insignificant as well.

Discussion

Community-acquired pneumonia (CAP) is a cause of substantial morbidity, mortality, and resource utilization worldwide [19]. In the RCTs included in our study, the diagnostic criteria for CAP were as follows: patients having fever and/or a WBC count >10,000/mm^3^, and/or a neutrophil count >70% and at least three of the following symptoms - cough, purulent sputum, dyspnea or tachypnea, chest pain, evidence of pulmonary consolidation. Only the cases of the patients suitable for being managed with outpatient therapy with an oral antimicrobial agent were considered and included. Despite the advancements in the treatment of CAP, antimicrobial management is still controversial [20]. The antibiotics used in the RCTs were confirmed with the guidelines of the Infectious Disease Society of America (IDSA)/American Thoracic Society (ATS) [21] and the Canadian Infectious Disease Society (CIDS)/Canadian Thoracic Society (CTS). After an initial assessment of severity, the patients classified as having mild to moderate cases were managed orally. 

Our primary outcome analysis based on four RCTs [14-17] validates that nemonoxacin and levofloxacin are parallel concerning mortality reduction and clinical response in the treatment of CAP. Notably, the clinical cure rates of nemonoxacin were on par with levofloxacin in CAP patients. In the same way, the clinical failure rates were also indistinguishable. Furthermore, both dosages (500 mg and 750 mg) of nemonoxacin were found to be comparable with levofloxacin (750 mg). Lastly, the clinical efficacy of nemonoxacin was analogous to levofloxacin in the treatment of CAP. This meta-analysis aims to compare the efficacy as well as the safety of nemonoxacin and levofloxacin. Our efficacy findings are supported by a previous meta-analysis [22] that evaluated the same antibiotics for the treatment of CAP. However, the previous meta-analysis was based on three RCTs, while this updated meta-analysis is based on four RCTs. Moreover, the previous meta-analysis has mentioned safety concerns as the primary outcome and microbiological response as the secondary outcome [22]. Alternatively, this is the only detailed meta-analysis focused on the safety and efficacy of nemonoxacin and levofloxacin. Three [14-16] out of four of our included RCTs have mentioned the safety and efficacy along with the microbiological response acquired from these drugs. On the other hand, Cheng et al. [17] have focused mainly on the clinical cure rates, that is, the safety concerns associated with quinolones. 

The adverse reactions of the intervention mainly involve gastrointestinal, hepatic, cardiac, hematologic, and neurological-related symptoms [23]. It was observed that the most frequent adverse event in the gastrointestinal system was nausea, as reported by all four RCTs [14-17]. Vomiting and epigastric pain were reported by three RCTs and occurred almost evenly in both groups. Diarrhea, nonetheless, was reported by two RCTs [14,17] but was more commonly reported by the nemonoxacin group. It was observed that diarrhea and nausea were drug-related TEAEs, and, therefore, were resolved with the discontinuity of the drug. Henceforth, it is suggested to manage the preventive workup at the earliest possible interval. The overall results, however, favored the safety of the drug. Similar to other quinolones, hepatotoxic side effects, which consist mainly of elevated AST, ALT, and liver function tests (LFTs), were not found to be reported as drug-related TEAE, although a larger, but not significant, increase of ALT was noted in patients treated with nemonoxacin.

The cardio-toxic side effects include QT interval prolongation and bundle branch blockage [24]. According to Pharmacovigilance Working Party (PhVWP) [25], some fluoroquinolones can cause torsade's de points, especially those that favor QT interval prolongation. When reviewed for safety in this aspect, it was concluded that the fluoroquinolone levofloxacin had a low potential of causing this outcome with no reported cases of torsade's de points [25]. Regarding the bundle branch blockage, only four patients from the nemonoxacin group reported it, contrary to the levofloxacin group, where no such findings were observed. It is recommended to consider the actual condition of the patient's body thoroughly so that there is an immediate adjustment of the medication regimen or stoppage of the treatment [26]. The hematologic adverse events consist of drug-related TEAE, which includes neutropenia and thrombocytopenia, and common TEAE, which includes leukopenia and skin rash. No such effects were significantly reported by either of the groups. Lastly, a few neurological symptoms, mainly dizziness and headache, were also reported by some patients in various studies [27]. 

We found a few differences in the drug-related TEAE. The most significant difference was found concerning intestinal events. In contrast to Cheng et al. [17] study, which reported more cases of drug-related diarrhea in the nemonoxacin group and, therefore, favored levofloxacin, the study by van Rensburg et al. [14] reported more such cases in the levofloxacin group, hence favoring nemonoxacin. However, as only two studies have reported diarrhea [14,17] as a drug-related TEAE with variable outcomes, we can conclude that this may have been an outcome of high-observed heterogeneity and, hence, have precluded the detection of this side effect with levofloxacin. 

There is an association found between nausea and thrombocytopenia in patients treated with nemonoxacin [28]. However, when compared with levofloxacin, we deduced that it was more frequent in nemonoxacin. Neutropenia was seen in the nemonoxacin group, but a significant conclusion cannot be procured as individual differences between the 1260 patients were minimal. Finally, the overall drug-related TEAE further affirms a non-significant difference in the drug safety profiles. 

The core strength of our research is that it is solely based on RCTs; therefore, there is a reduced risk of bias. Moreover, this updated meta-analysis has focused mainly on safety and efficacy by analyzing the TEAEs of the respective drugs. Furthermore, the robustness of this meta-analysis can be considered more convincing than individual RCTs. Lastly, this meta-analysis can resolve the dissimilitude between the studies, which can help in yielding conclusive results for the greater good of pharmacotherapy in patients suffering from CAP. 

Conversely, there are certain limitations to this study. Firstly, a limited number of studies were included in this meta-analysis; therefore, the risk of heterogeneity may be underestimated. Secondly, the results of this analysis cannot be generalized to the unselected population due to the narrow inclusion criteria of RCTs. In addition, the degree of severity of CAP in this study was mild to moderate. Therefore, further studies are required to evaluate the use of nemonoxacin in severe CAP. Thirdly, this meta-analysis is based on the use of oral nemonoxacin. Further investigations are required for the safety and efficacy of intravenous nemonoxacin.

## Conclusions

Succinctly, 500 mg or 750 mg of oral nemonoxacin administered once daily for seven to 10 days showed robust clinical efficacy in treating adult CAP. Moreover, although nearly all treatment-emergent adverse events are higher in the nemonoxacin group, statistically, we should consider it equally well tolerated as levofloxacin because the differences are not significant. Thus, nemonoxacin can be recommended as an appropriate antibiotic therapy for mild to moderate CAP.

## Appendices

**Table 4:** Detailed search strategy

Database	Search strategy	Results
PubMed	((((nemonoxacin) OR (quinolone)) OR (non- fluorinated quinolone)) AND ((levofloxacin) OR (ofloxacin))) AND (((community-acquired pneumonia) OR (pneumonia)) OR (CAP))	1717
Google Scholar	((((nemonoxacin) OR (quinolone)) OR (non- fluorinated quinolone)) AND ((levofloxacin) OR (ofloxacin))) AND (((community-acquired pneumonia) OR (pneumonia)) OR (CAP))	154
Scopus	((((nemonoxacin) OR (quinolone)) OR (non- fluorinated quinolone)) AND ((levofloxacin) OR (ofloxacin))) AND (((community-acquired pneumonia) OR (pneumonia)) OR (CAP))	104

**Table 5:** Study characteristics of randomized control trials (RCTs)

Study name	Van Rensburg et al. (2010) [14]	Liu et al. (2015) [15]	Yuan et al. (2017) [16]	Cheng et al. (2018) [17]
Study title	Efficacy and safety of nemonoxacin versus levofloxacin for community-acquired pneumonia	A randomized, double-blind, multicenter Phase II study comparing the efficacy and safety of oral nemonoxacin with oral levofloxacin in the treatment of community-acquired pneumonia	Safety and efficacy of oral nemonoxacin versus levofloxacin in treatment of community-acquired pneumonia: A phase 3, multicenter, randomized, double-blind, double-dummy, active-controlled, non-inferiority trial	Integrated safety summary of phase II and III studies comparing oral nemonoxacin and levofloxacin in community-acquired pneumonia
Patients enrollment, n	265	192	527	995
Start date of trial	N/A	August 2009	April 2011	December 2006
End of trial	N/A	August 2010	December 2012	August 2012
Year of trial	2010	2010	2012	2012
Duration of trial	7 days	7-10 days	7-10 days	3-10 days
Phase of RCT	2	N/A	3	2, 3
Trial type	Randomized, double-blind, multicenter	A phase II, randomized, double-blind, double-dummy, multicenter	N/A	N/A
Population included	An oral temperature of at least 38°C or equivalent tympanic or rectal temperature within 24 hours was required for enrollment in those over the age of 18, as chilled, shortness of breath, tachypnea (more than 20 breaths per minute), cough, pleuritic chest pain, purulent sputum, and a fever. A radiologist verified patients with new or persistent/progressive infiltrates on chest radiographs.	To be eligible, patients must be between 18 and 70, have a body mass index (BMI) of at least 18 kg/m2, weigh between 40 and 100 kg, and have mild to moderate community-acquired pneumonia (CAP). Two or more symptoms confirmed CAP, and a chest X-ray taken within 48 hours showed new lobar or multilobar infiltrates. Infection symptoms include a high body temperature (oral temperature of at least 37.3°C), coughing up purulent sputum, lung consolidation, and rales, and a peripheral white blood cell count of more than 10109 cells/L or less than 4 109 cells/L, or a neutrophil level of more than 70%.	A minimum of three symptoms were required for inclusion: cough, purulent sputum, dyspnea or tachypnea, chest discomfort, and indications of lung consolidation in patients aged 18 to 70 with community-acquired pneumonia (CAP). As an outpatient, also need to take antibiotics by mouth. Individuals were only eligible if they had experienced pulmonary exudation or infiltration within the past 48 hours. There needed to be at least 25 leukocytes and fewer than ten squamous epithelial cells in the sputum sample.	Participants were eligible if they were 18 or older, of either gender, diagnosed with CAP, and stable enough to receive treatment in an outpatient setting. New or persistent infiltrates on chest x-ray for more than 48 hours following therapy were inclusion criteria.
Inclusion criteria	Patients were considered for inclusion if they met the following criteria for a diagnosis of mild to moderate community-acquired pneumonia (CAP): fever (oral temperature of 38°C or greater, or equivalent tympanic or rectal temperature) and at least one of the following symptoms: chills, shortness of breath, tachypnea (greater than 20 breaths/minute), cough, pleuritic chest pain, purulent sputum, Crohn's disease. Participation also necessitated a chest X-ray that was confirmed by a radiologist and showed either new or ongoing infiltrates.	Involvement was open to patients with mild to severe CAP with a body mass index (BMI) of less than 18. In the past, a diagnosis of CAP was made with the presence of new lobar or multilobar infiltrates consistent with CAP within 48 hours of a chest x-ray, a worsening of respiratory symptoms, evidence of pulmonary consolidation and rales, a peripheral white blood cell count of >10 109 cells/L or >4 109 cells/L, or a neutrophil level >70%.	To be eligible for enrollment, a patient had to be between the ages of 18 and 70, have a clinical diagnosis of community-acquired pneumonia (CAP) with fever or a white blood cell (WBC) count of >10,000/mm3 or a neutrophil count of >70%, have at least three of the following symptoms: cough, purulent sputum, dyspnea or tachypnea, chest pain, and evidence of pulmonary consolidation, be suitable for outpatient therapy.	Male or female participants aged 18 and up with a confirmed diagnosis of CAP who are medically stable enough for outpatient care are eligible to participate. Chest radiographs must reveal new or continuing/progressive infiltrates within 48 hours of the first medicine dosage for enrollment to occur.
Exclusion criteria	Females, either expecting a child or nursing one, were excluded from the research. Patients with a history of anaphylaxis to or hypersensitivity to any quinolone, fluoroquinolone tendinopathy, chronic renal failure, clinically significant hepatic disease, hematological malignancy, immunodeficiency (such as neutropenia), prolonged electrocardiogram QT corrected (QTc) interval, or the need for concomitant medication associated with increased QTc interval, clinically significant conduction, or other abnormality on a 12-le Individuals with malignant lung diseases were excluded, as were those with severe cases of bronchiectasis, cystic fibrosis, active tuberculosis, bronchial obstruction, post-obstructive pneumonia, or a septic pleural effusion. Participants who had received antibiotics within seven days of enrollment or any other investigational drug within one month before randomization were ineligible, as were those who abused alcohol or drugs, had received chemotherapeutic agents or oncolytic within the six months before randomization, or expected to require such agents during the course of the study. After three days of treatment, the medication could be discontinued if the patient's symptoms had not improved or had worsened. If therapy for CAP became necessary, the researcher might decide to use an antibiotic other than quinolone.	Those with active forms of tuberculosis, bronchiectasis, lung cancer, renal failure, hepatic dysfunction, or a history of mental illness were not included. Patients with a history of anaphylaxis, an allergy to quinolones, chemotherapy, or oncolytic during the previous six months, or a history of substance misuse were not permitted. Ineligible participants required combination antimicrobial medication due to dual infection, had gotten any quinolone two weeks before randomization, had received 500 mL of blood in the three months before enrollment, or had taken any investigational drug. Finally, abnormal cardiac conduction or prolonged QT intervals (QTc >430 ms in males and >450 ms in females) were ruled out by 12-lead electrocardiograms. Participants could be disqualified if their symptoms worsened or didn't improve after three days. The study's doctor might try treating CAP with an antibiotic other than quinolone.	Patients could not enroll if they had septic shock, acute pneumonia, or at least three other disorders. Non-diabetic hypoglycemia, acute alcoholism, hyponatremia (Na+ 130 mM), metabolic acidosis of unknown origin or elevated lactic acid, liver cirrhosis, asplenia syndrome, respiratory rate of 30 breaths per minute, multilobar infiltrates on chest radiograph, a disorder of consciousness; hypotension requiring fluid resuscitation; multilobar infiltrates on chest radiograph; disorder of consciousness. Patients were not eligible if they had been hospitalized within 14 days before enrollment if they had lung disease, QTc prolongation, severe cardiac insufficiency, epileptic seizure activity, hypersensitivity to any quinolone, fluoroquinolone tendinopathy, myasthenia gravis, or if their screening 12-lead electrocardiogram (ECG) showed arrhythmias. Women who were pregnant or nursing were not permitted.	Active or chronic pulmonary illness other than CAP, clinically significant hepatic or renal disease, central nervous system diseases, uncontrolled mental problems, malignancies, or immunodeficiency were excluded from the study. Subjects who were pregnant or nursing, had taken antibiotics within the previous week, or had a lung illness other than CAP were also excluded.
Regimens/treatment	Those who qualified were given either 750 mg of nemonoxacin, 500 mg of nemonoxacin, or 500 mg of levofloxacin orally once a day for seven days. The investigational drug's initial dose was given in the clinic under their supervision. When patients fasted for 10 hours, they self-administered multiple doses with water. After taking the research medication, participants might drink up to a quart (240 mL). They had to wait two hours without eating, in any case.	Participants who met the inclusion criteria were given either 500 mg of nemonoxacin, 750 mg of levofloxacin, or a 500 mg oral placebo. During 7-10 days, the medicine was taken once daily. After the patient joined up, the first dose was given to them at the clinic, where a doctor could closely monitor them. After that, it was up to the patients to provide their morning doses.	Using block randomization with an IWS, patients were assigned a 2:1 chance of receiving nemonoxacin or levofloxacin. The recommended dosage for both medications was 500 mg, taken orally once daily for 7-10 days. At least three days of treatment were given to each patient before any conclusions could be drawn about the treatment's success. Participants were evaluated four times: before treatment (within 24 hours of the first dose), during treatment (days 41), at the end of therapy (days 1-2 after the last dose), and as a test of cure (7-14 days post-therapy or early-termination).	In two studies, participants took the study medication orally once daily for 7-10 days. In the third, they took it for only seven days. Following the end of the therapy phase, the participants were followed up for another 14 days.
Primary outcomes	The clinical response at the TOC or ET visit served as the primary efficacy outcome of the trial.	The primary efficacy endpoint of this trial was clinical response at the test of cure (TOC) visit, which occurred 7-10 days after treatment ended. Clinical cure was defined as the complete resolution of all pneumonia symptoms or recovery to the pretreatment state; clinical failure was defined as the persistence or worsening of symptoms after therapy, the emergence of new pneumonia-related symptoms or signs, and the use of other antimicrobial therapy targeting pneumonia; and early withdrawal from the study due to an adverse event was considered a "clinical failure."	Most patients (>80%) experienced mild drug-related AEs, and the severity was comparable among treatment groups. The most common adverse effects (AEs) were neutropenia, nausea, leukopenia, and increased alanine aminotransferase.	Most patients (>80%) experienced mild drug-related AEs, and the severity was comparable among treatment groups. The most common adverse effects (AEs) were neutropenia, nausea, leukopenia, and increased alanine aminotransferase. Most patients (>80%) experienced mild drug-related AEs, and the severity was comparable among treatment groups. The most common adverse effects (AEs) were neutropenia, nausea, leukopenia, and increased alanine aminotransferase. Most patients (>80%) experienced mild drug-related AEs, and the severity was comparable among treatment groups. The most common adverse effects (AEs) were neutropenia, nausea, leukopenia, and increased alanine aminotransferase.
Secondary outcomes	The secondary efficacy variable of this study was the bacteriological response. At the pretreatment visit (day -1), sputum samples were collected by expectoration after deep coughing.	The trial's secondary objectives were clinical and bacteriological response rates, assessed at the end of therapy and the test-of-cure (TOC) visit, respectively, in both the complete analysis set (FAS) and the per-protocol set.	The secondary outcomes were either positive or negative. Per-pathogen clinical and microbiological response, safety, TOC, end-of-therapy (EOT) b-mITT, and bacteriologically evaluable (BE) microbiological efficacy rates were measured. Clinically evaluable (CE) patients had TOC and EOT cure rates.	N/A
