# Correction: Safety and Efficacy of Nemonoxacin vs Levofloxacin in Patients With Community-Acquired Pneumonia: A Systematic Review and Meta-Analysis of Randomized Control Trials

**DOI:** 10.7759/cureus.c305

**Published:** 2025-09-23

**Authors:** Alina S Khan, Arham Iqbal, Alina A Muhammad, Fariha Mazhar, Muniba F Lodhi, Komal F Ahmed, Satesh Kumar, Giustino Varrassi, Mahima Khatri

**Affiliations:** 1 Medicine and Surgery, Liaquat National Hospital and Medical College, Karachi, PAK; 2 Medicine and Surgery, Dow University of Health Sciences, Dow International Medical College, Karachi, PAK; 3 Medicine and Surgery, Ziauddin University, Karachi, PAK; 4 Medicine and Surgery, Shaheed Mohtarma Benazir Bhutto Medical College, Karachi, PAK; 5 Pain Medicine, Paolo Procacci Foundation, Rome, ITA; 6 Medicine and Surgery, Dow University of Health Sciences, Karachi, Karachi, PAK

This article has been corrected to fix the following typos in the PRISMA diagram: 

- Records removed before screening corrected from 2,125 to 1,425
- Reports excluded due to "full text not available, retraction, or erroneous indexing" corrected from 0 to 10

